# Liver phospholipids fatty acids composition in response to different types of diets in rats of both sexes

**DOI:** 10.1186/s12944-017-0483-9

**Published:** 2017-05-19

**Authors:** Slavica Ranković, Tamara Popović, Jasmina Debeljak Martačić, Snježana Petrović, Mirko Tomić, Đurđica Ignjatović, Gordana Tovilović-Kovačević, Maria Glibetić

**Affiliations:** 1 Centre of Research Excellence in Nutrition and Metabolism, Institute for Medical Research, University of Belgrade, Tadeuša Košćuška 1, Belgrade, 11129 Serbia; 2 Institute for Biological Research “Siniša Stanković”, University of Belgrade, Bulevar despota Stefana 142, Belgrade, 11060 Serbia

**Keywords:** Fish based diet, Milk based diet, Fatty acids, Gender, Rats

## Abstract

**Background:**

Dietary intake influence changes in fatty acids (FA) profiles in liver which plays a central role in fatty acid metabolism, triacylglycerol synthesis and energy homeostasis. We investigated the effects of 4-weeks treatment with milk- and fish-based diet, on plasma biochemical parameters and FA composition of liver phospholipids (PL) in rats of both sexes.

**Methods:**

Adult, 4 months old, Wistar rats of both sexes, were fed with different types of diets: standard, milk-based and fish-based, during 4 weeks. Analytical characterization of different foods was done. Biochemical parameters in plasma were determined. Fatty acid composition was analyzed by gas-chromatography. Statistical significance of FA levels was tested with two-way analysis of variance (ANOVA) using the sex of animals and treatment (type of diet) as factors on logarithmic or trigonometric transformed data.

**Results:**

Our results showed that both, milk- and fish-based diet, changed the composition and ratio of rat liver phospholipids FA, in gender-specific manner. Initially present sex differences appear to be dietary modulated. Although, applied diets changed the ratio of total saturated fatty acids (SFA), monounsaturated fatty acids (MUFA) and polyunsaturated fatty acids (PUFA), and effects were gender specific. Milk-based diet lowered SFA and elevated MUFA in males and increased PUFA in females vs. standard diet. The same diet decreased n-3, increased n-6 and n-6/n-3 ratio in males. Fish-based diet increased n-3, decreased n-6 and n-6/n-3 ratio vs. standard and milk-based diet in females. However, the ratio of individual FA in liver PL was also dietary-influenced, but with gender specific manner. While in females fish-based diet decreased AA (arachidonic acid) increased level of EPA (eicosapentaenoic acid), DPA (docosapentaenoic acid) and DHA (docosahexaenoic acid), the same diet elevated only DHA levels in males.

**Conclusion:**

Gender related variations in FA composition of rat liver PL were observed, and results have shown that those initial differences could be significantly modulated by the type of diet. Furthermore, the modulatory effects of milk- and fish-based diets on liver phospholipids FA profiles appeared to be sex-specific.

## Background

Phospholipids (PL) synthesis and metabolism were found to take place predominantly in hepatic tissue [1]. The fatty acids (FA) profile of liver PL and triglycerides (TG) is known to be influenced by many factors, including dietary intake, age, gender and endogenous metabolism [2]. FA plays a role in the modulation of membrane fluidity, interacts with intracellular signaling pathways and act as substrates for the production of signaling molecules [3].

Dietary intake influence changes in FA profiles and endogenous metabolism in different tissues. The balance between total n-6 and n-3 polyunsaturated fatty acids (PUFA) in the diet is important because of their competitive nature.

Increased consumption of oily fish or fish oil supplements reach in n-3 PUFA has been shown to increase plasma, cell and tissue eicosapentaenoic acid (EPA) and docosahexaenoic acid (DHA) concentrations in human, which is associated with health benefits, particularly in relation to cardiovascular and inflammatory diseases, and brain function [4, 5]. Consumption of fish oil significantly increased EPA and n-3 docosapentaenoic acid (DPA) in liver PL of male rats [6]. On the other side, the milk-fat-based diet which had both, significant contributions of saturated fatty acids (SFA) and low PUFA content, also reflected on plasma and tissue FA composition in rats [7].

Gender related differences in FA composition of different lipid classes are well established. Arachidonic acid (AA) and DHA proportions are higher in liver and plasma PL in female than male rats fed on standard chow diet [8]. Female rats replete their DHA status more readily than males, probably due to a higher expression of liver desaturases which should be taken into account for specific nutritional recommendations [9]. In liver PL, females exhibited higher percentages of DHA, EPA, and overall n-3, while the percentage of DPA was lower, together with higher hepatic ∆-6 desaturase mRNA and protein content, which suggests that females have a higher capacity to synthesize DHA from shorter chain n-3 PUFA than males. This expression difference seems to be limited to the liver [10].

The aim of the present study was to analytically characterize and examine the effects of different diets, fish-based and milk-based vs. standard diet, on plasma biochemical parameters and PL fatty acids composition in rats liver of both sexes.

## Methods

### Laboratory animals and treatments

Adult Wistar rats of both sexes, provided by the vivarium of the Institute for Biological Research (Belgrade, Serbia), were used. The animals were kept in groups of 4 per cage, under controlled conditions (room temperature 23–25 °C, 12 h light-dark cycle, drinking liquid ad libitum). The overall maintenance of animals and experimental protocols were in accordance to the Official Institutional Guide for Experimental Work on Animals, adjusted to the European Communities Council Directive (86/609) and the Guide for Care and Use of Laboratory Animals, NIH publication No. 85–23. Animals were randomly assigned in three groups of both sexes, 4 months old, weighing 250–300 g. Bodyweight of rats was registered weekly and presented as percentage of body mass increase at the end of the study for each experimental group. The values were as follows: males fed on standard diet (MS), males fed on fish based diet (MF), males fed on milk based diet (MM): (10.1 ± 0.7, 6.3 ± 0.2, 13.0 ± 1.5) and females fed on standard diet (FS), females fed on fish based diet (FF), females fed on milk based diet (FM): (7.2 ± 0.8, 4.4 ± 0.1, 8.1 ± 0.7). Control group was fed with standard laboratory diet, milk-based diet group and fish-based diet group during 4 weeks treatment. Feed intake was the same in all three experimental groups and there was no statistically significant change in daily food consumption.

### Rat chow

The composition of rat chow was established on a prescription from the department of Animal Nutrition and Botany (Faculty of Veterinary Medicine, University of Belgrade). The whole stock of the rat chow is prepared in the laboratory by mixing the components obtained from the commercial suppliers (“Farmcommerce”, Čantavir, Serbia; “Granexport”, Pančevo, Serbia). The chow mixture was made of ground cereals: barley (30%), corn (13%) and wheat (20%); dried sugar-beet pulp shred (10%), 1% of the mineral mixture (20% CaHPO4·2H2O, 30% CaCO3, 50% NaCl), and 1% of the vitamin mixture (contains *per* kg of vitamin mixture: retinol 186 mg, cholecalciferol 20 μg, alpha-tocopherol acetate 10 g, menaquinone 4 g, thiamine 1.2 g, riboflavin 0.6 g, pyridoxine 1.5 mg, cyancobalamine 4 mg, niacine 3.6 g, pantothenic acid 1.9 g, biotine 12 mg, choline 101.2 g, inositol 93 g; prepared upon request by the Veterinary Institute, Subotica, Serbia). The main source of proteins in the chow were either 9% of fishmeal (anchovy; “Century-Light Industry Co.“, Weifang, China) or 10% of dried milk powder (“AD Mlekara”, Subotica, Serbia), and 16% and 15% of soybean meal (“Sojaprotein”, Bečej, Serbia), respectively. The final solid rat chow was prepared once weekly by stirring the chow mixture with tap water (m/m ratio 5:3) in the appropriate electrical blender. Each dough was outstretched in a thin layer (1 cm), pre-cut (3 cm × 3 cm) and dried for 30 h at 50 °C. The parched pieces of solid chow resulted in up to 10% of moisture.

### Analytical analysis of diets

Nutritional analysis was carried out by two accredited laboratories. Total carbohydrates (TCH) content, crude “by difference”, was calculated by the following formula: TCH (%) = 100% - % (CP + A + CF + M). Ash content was determined by direct gravimetric method (AOAC 923.03) that includes ashing of the samples in an oven at 550 °C until constant weight was attained. Crude proteins (CP) content was estimated based on total nitrogen content of samples (determined by Kjeldahl method (AOAC 955.04D). Crude fat content was determined gravimetrically (Soxhlet extraction, AOAC method (AOAC 963.15).Energy values of selected composite food were calculated based on determined content by the following formula: Energy value (estimated, kJ/100 g) = [4 x protein (%)] + [4 x carbohydrate (%)] + [9 x fat (%)]. Microelements were determined by graphite furnace atomic absorption spectrometric technique (GFAAS), reference document, EPA 200.9 (1994). Vitamin A and vitamin E were determined by HPLC (Table 1). (Agilent Technologies 1200 Series with DAD detector), on a column Agilent ZORBAX C18 (2.1 × 100) 1.8 μm. Mobile phase: methanol (100% HPLC purity). Macro and microelements were determined by method AAS (Varian spectra AA**-**10) (Table 1).

**Table 1:** Determination of contents of rat chow

	Standard diet	Fish based diet	Milk based diet
Proteins %	20	21.15	16.35
Fats %	4.2	2.42	4.07
Cellulose %	8	4.44	3.51
Starch %	38	40.31	36.86
Ash %	10	5.54	4.16
Energy KJ/100 g	1100	1128.51	1105.5
Carbohydrates %	3.3	1.49	4.43
Calcium %	1	1.25	0.98
Phosphor %	0.5	0.12	0.07
Sodium mg/kg	0.25	0. 3242	0. 2503
Magnesium mg/kg	1000	1673	1586
Iron mg/kg	100	126.2	88.12
Zn mg/kg	100	35.2	27.11
Vit E mg/kg	25	54.96	23.1
Vit A IU/kg	10	29.86	24.25

For analysis of FA composition, the fats are extracted from the sample with supercritical extraction CO_2_ at FAT 2000 analyzer (Leco, St. Joseph, MI, USA), in order to prevent the chemical degradation of the FA, or the extraction of the aggressive conditions. As a solvent n-heptane was used, for initiation and release of FA methyl esters from the residue of the solvent evaporation was applied to a stream of nitrogen. Prepared samples were analyzed with gas chromatography (GC) Agilent 7890A system (Agilent Technologies, Santa Clara, CA, USA) with a FID (Flame Ionization Detector), auto-injection mode for liquid equipped with fused silica capillary column (DB-WAX 30 m, 0.25 mm, 0.50 μm. The FA peaks were identified by comparing retention times of samples with retention times of standards “Supelco 37 component FA acid methyl ester mix” as well as internal data obtained in previous studies of FA on gas chromatography with mass detector. The results were expressed as mass of FA or a fatty acid group (g) in 100 g of FA (Table 2).

**Table 2:** Fatty acids profiles and overall SFA, MUFA, PUFA in different types of diet

Fatty acids (%)	Standard food	Fish based diet	Milk based diet
C6:0		2.769	1.627
C8:0			0.656
C10:0			1.5109
C11:0			0.173
C12:0	0.05	0.0417	1.813
C14:0	0.51	1.5030	6.344
C14:1			0.539
C15:0	0.08	0.1846	0.724
C16:0	13.55	15.555	25.36
C16:1	1.04	1.5731	1.585
C17:0		0.0356	0.543
C17:1		0.092	0.197
C18:0	2.30	3.394	7.028
C18:1n-9	24.95	20.798	
C18:1n-9 t			1.34
C18:1n-9c			21.54
C18:2n-6	49.93	38.955	23.458
C20:0		0.414	0.451
C18:3n-3	1.17	1.434	2.012
C18:3n-6	0.19		
C20:1	3.25	1.548	
C21:0		3.249	0.33
C20:2	0.13	0.5935	0.036
C20:4n-6			1.00
C22:0		0.2835	0.198
C22:1n-9		1.8511	0.233
C20:3n-3	0.72		
C20:3n-6	0.12		0.995
C23:0		0.322	0.289
C24:0	1.38		
C20:5n-3	0.06	2.4738	
C22:6n-3	0.58	2.928	
SFA	18.91	27.75	47.05
MUFA	29.24	25.86	25.43
PUFA	52.9	46.38	27.50

### Analyzing of biochemical parameters

Blood samples (6–8 cm^3^) from all rats were obtained after the end of intervention via *aorta abdominalis* puncture and collected in tubes containing Na-citrate (3.8% *w*/*v*) as anticoagulant. Plasma samples were collected upon centrifugation. Biochemical parameters in plasma were determined using standard laboratory kits (Roche) on biochemical analyzer (Cobas c-111, Roche, Basel, Switzerland). Parts of the animal livers were frozen at −80 °C.

### Fatty acids analyzing in liver phospholipids

The method consists of homogenizing the liver tissue with a 2:1 chloroform/methanol mixture [6]. The PL fraction was isolated from the extracted lipids by one-dimensional thin liquid chromatography (TLC) neutral lipid solvent system of hexane:diethyl-ether:acetic acid (87:2:1) using Silica Gel GF plates (C. Merck, Darmstadt, Germany). FA methyl esters derivatives formed from isolated liver PL fraction were separated by Gas Chromatography using Shimadzu GC 2014 equipped with a flame ionization detector and DB-23 fused silica gel capillary column. Comparing sample peak retention times with authentic standards (Sigma Chemical Company) and/or the (PUFA)-2 standard mixtures (Restec) identified individual FA methyl esters.

### Statistical analysis

Statistical analyses were performed according to Hinkle et al. [11] and Manly [12]. Data are expressed as the means ± SE. Statistical significance of FA levels was tested with two-way analysis of variance (ANOVA) using the sex of animals and treatment (type of diet) as factors on logarithmic or trigonometric transformed data. The trends were considered as significant if *p* < 0.05. The values were post hoc compared by Turkey’s HSD test. The composition of FA in liver of rats fed on different diet was compared by canonical discriminant analysis.

## Results

**Fig. 1:**
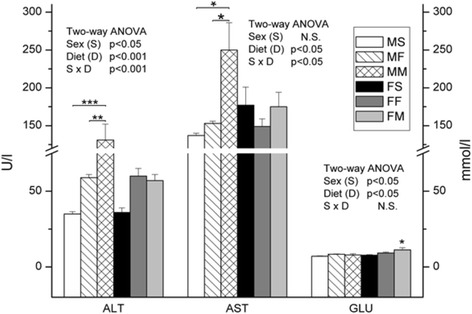
ALT and AST activities and the concentration of glucose in plasma. Data are given as mean ± SE (*n* = 6). Results were tested by two-way ANOVA with sex (S) and diet (D) factors (*p* significance was showed) and post hoc tested by Tukey’s HSD. *** - *p* < 0.001, ** - *p* < 0.01, * - *p* < 0.05. MS – males fed on standard diet, MF – males fed on fish based diet, MM – males fed on milk based diet, FS – females fed on standard diet, FF – females fed on fish based diet, and FM – females fed on milk based diet

Milk-based diet only slightly elevated glucose level (*p* < 0.05) in females, but significantly alanine aminotransferase (ALT) (*p* < 0.001) and aspartate aminotransferase (AST) (*p* < 0.05) activities in males comparing to standard diet. Fish based diet had no significant effects on ALT and AST levels, as well as glucose levels in both females and males (Fig. 1).

**Fig. 2:**
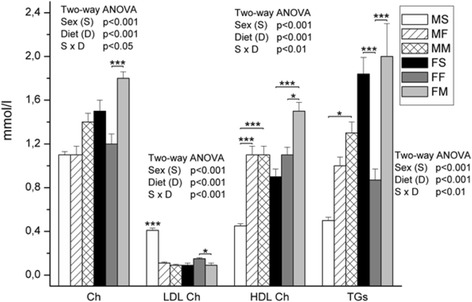
The amount of cholesterol, LDL cholesterol, HDL cholesterol and triglicerides in the blood plasma. Data are given as mean ± SE (*n* = 6). Results were tested by two-way ANOVA with sex (S) and diet (D) factors (*p* significance was showed) and post hoc tested by Tukey’s HSD. *** - *p* < 0.001, ** - *p* < 0.01, * - *p* < 0.05. MS – males fed on standard diet, MF – males fed on fish based diet, MM – males fed on milk based diet, FS – females fed on standard diet, FF – females fed on fish based diet, and FM – females fed on milk based diet

In females, milk based diet elevated the amount of HDL cholesterol (*p* < 0.001) (Fig. 2) comparing to controls. However, fish based diet lowered the TG (*p* < 0.001). In males, both fish- and milk-based types of diet lowered low-density lipoprotein (LDL) (*p* < 0.001) and increased HDL (*p* < 0.001) cholesterol comparing to standardized diet. Milk based diet elevated TG (*p* < 0.05).

**Fig. 3:**
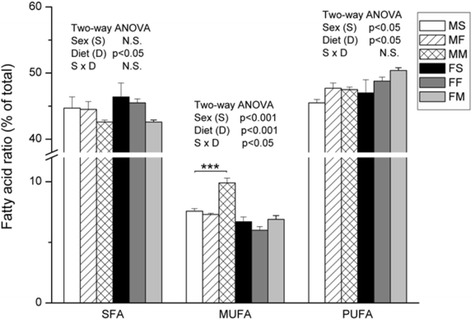
Saturated fatty acids, monounsaturated fatty acids and polyunsaturated fatty acids percentage in liver. Data are given as mean ± SE (*n* = 6). Results were tested by two-way ANOVA with sex (S) and diet (D) factors (*p* significance was showed) and post hoc tested by Tukey’s HSD. *** - *p* < 0.001, ** - *p* < 0.01, * - *p* < 0.05. MS – males fed on standard diet, MF – males fed on fish based diet, MM – males fed on milk based diet, FS – females fed on standard diet, FF – females fed on fish based diet, and FM – females fed on milk based diet

Diet changes the ratio of SFA, MUFA and PUFA (Fig. 3, significant ANOVA diet effect), but the effects were sex specific. Milk based diet lowered the amount of SFA (*p* < 0.05) and elevated the ratio of MUFA (ANOVA diet effect, *p* < 0.001) in males (ANOVA sex effect *p* < 0.001, and interaction S x D, *p* < 0.05), and PUFA, in females (ANOVA sex effect *p* < 0.05, and diet effect *p* < 0.05) comparing to standard diet.

**Fig. 4:**
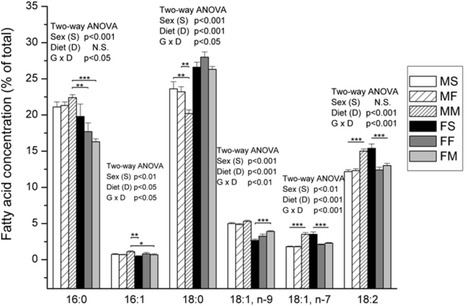
The percentage of palmitic acid, palmitoleic, stearic, oleic vaccenic and linoleic acid in liver. Data are given as mean ± SE (*n* = 6). Results were tested by two-way ANOVA with sex (S) and diet (D) factors (p significance was showed) and post hoc tested by Tukey’s HSD. *** - *p* < 0.001, ** - *p* < 0.01, * - *p* < 0.05. MS – males fed on standard diet, MF – males fed on fish based diet, MM – males fed on milk based diet, FS – females fed on standard diet, FF – females fed on fish based diet, and FM – females fed on milk based diet

**Fig. 5:**
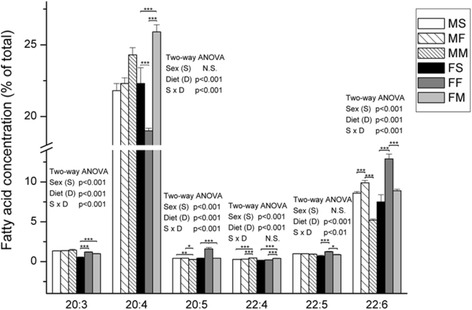
The percentage of ETA, AA, EPA, DTA, DPA and DHA in liver. Results were tested by two-way ANOVA with sex (S) and diet (D) factors (*p* significance was showed) and post hoc tested by Tukey’s HSD. *** - *p* < 0.001, ** - *p* < 0.01, * - *p* < 0.05. MS – males fed on standard diet, MF – males fed on fish based diet, MM – males fed on milk based diet, FS – females fed on standard diet, FF – females fed on fish based diet, and FM – females fed on milk based diet

Females had lower levels of palmitic acid (PA), palmitoleic and oleic acids (OA), and ETA (Figs. 4 and 5) as well as increased levels of stearic acid (SA) comparing to males (Fig. 4, significant ANOVA effect of sex). However, manipulation of diet type led to different changes in both males and females (ANOVA, S x D effect, *p* < 0.001).

Diet based on both fish and milk lowered the amount of vaccenic acid (VA) (*p* < 0.001) and linoleic acids (LA) (*p* < 0.001) in females liver phospholipids (Fig. 4). In females, milk based diet elevated oleic acid content (*p* < 0.001) (ANOVA significant effect of diet and interaction S x D, post hoc Tukey’s t-test).

On the other hand, in males, milk based diet elevated the amount of vaccenic (*p* < 0.001) and linoleic (*p* < 0.001) fatty acids comparing to other two types of diets (Fig. 4). Milk based diet also lowered the amount of SA in males (*p* < 0.01) (ANOVA significant effect of diet and interaction S x D, post hoc Tukey’s t-test).

In females, fish and milk based diet elevated ETA (*p* < 0.001) level comparing to standard diet (Fig. 5). Fish based diet lowered the amount of AA (*p* < 0.001) and increased levels of EPA (*p* < 0.001), DPA (*p* < 0.001) and DHA (*p* < 0.001) in relation to standard diet. Milk based diet elevated levels of AA (*p* < 0.001) and docosatetraenoic acid (DTA) (*p* < 0.001). In males, milk based diet decreased EPA (*p* < 0.01) and DHA (*p* < 0.001) and increased DTA (*p* < 0.001) levels compared to controls (Fig. 5). On the other hand, fish based diet elevated only DHA levels (*p* < 0.001) compared to standard diet (*p* < 0.001).

**Fig. 6:**
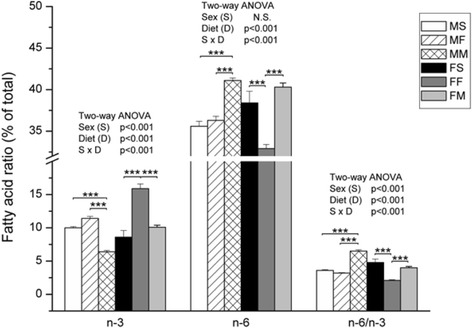
Fatty acids ratio (n-6/n-3) in liver of female and male rats. Data are given as mean ± SE (*n* = 6). Results were tested by two-way ANOVA with sex (S) and diet (D) factors (*p* significance was showed) and post hoc tested by Tukey’s HSD. *** - *p* < 0.001, ** - *p* < 0.01, * - *p* < 0.05. MS – males fed on standard diet, MF – males fed on fish based diet, MM – males fed on milk based diet, FS – females fed on standard diet, FF – females fed on fish based diet, and FM – females fed on milk based diet

Milk based diet decreased n-3 (*p* < 0.001), and increased n-6 (*p* < 0.001) and n-6/n-3 (*p* < 0.001) ratio in males comparing to the other type of diets. On the other hand, in females, fish based diet increased n-3 (*p* < 0.001), decreased n-6 (*p* < 0.001) and n-6/n-3 ratio (*p* < 0.001) comparing to standard and milk based diets (Fig. 6, ANOVA diet effect, *p* < 0.001, S x D effect, *p* < 0.001, Tukey’s post hoc).

**Fig. 7:**
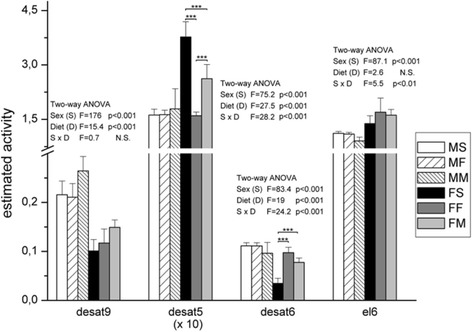
The estimated activities of desaturases 5, 6 and 9 and elongase in the liver. Data are given as mean ± SD. Results were tested by two-way ANOVA with sex (S) and diet (D) factors (F values and p significance are showed) and post hoc analyzed by Tukey’s HSD. *** - *p* < 0.001, ** - *p* < 0.01, * - *p* < 0.05. MS – males fed on standard diet, MF – males fed on fish based diet, MM – males fed on milk based diet, FS – females fed on standard diet, FF – females fed on fish based diet, and FM – females fed on milk based diet

Desaturase 9 activity was generally higher in males than females (sex effect in ANOVA, *p* < 0.001) (Fig. 7). However, in animals of both sexes fed with milk based diet, its activity was higher comparing to the other groups (diet effect in ANOVA, *p* < 0.001). On the other hand, females had higher levels of desaturase 5 comparing to males (sex effect in ANOVA, *p* < 0.001), when they were fed with standard and milk diet (diet effect, *p* < 0.001 and S x D effect, *p* < 0.001). Diet did not change the activity of desaturase 6 in males, but influenced its level in females (diet effect in ANOVA, *p* < 0.001, interaction S x D, *p* < 0.001). Females fed with standard diet had the lowest level of desaturase 6 (post hoc Tukey’s test, *p* < 0.001), but feeding with milk and fish based diet elevated its activity almost to the levels measured in males. Females had significantly higher levels of elongase 6 comparing to males (sex effect ANOVA, *p* < 0.001). There was no statistical significant effect of diet on the levels of elongase 6 in examined groups.

**Fig. 8:**
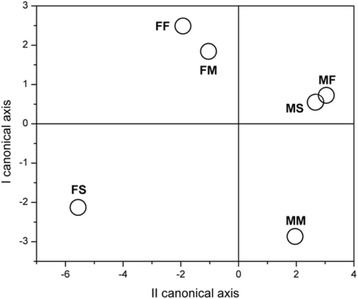
Canonical discriminant anlysis of SFA and MUFA composition in rats fed with different diet. Data are expressed as mean of canonical variables ± SE. MS – males fed on standard diet, MF – males fed on fish based diet, MM – males fed on milk based diet, FS – females fed on standard diet, FF – females fed on fish based diet, and FM – females fed on milk based diet

**Fig. 9:**
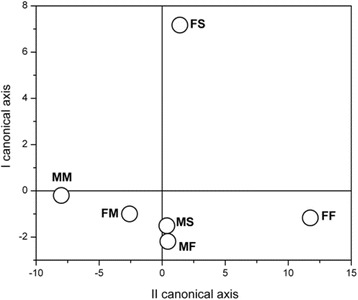
Canonical discriminant anlysis of PUFA composition in males and females fed with different diet. Data are expressed as mean of canonical variables ± SE. MS – males fed on standard diet, MF – males fed on fish based diet, MM – males fed on milk based diet, FS – females fed on standard diet, FF – females fed on fish based diet, and FM – females fed on milk based diet

Canonical discriminant analysis showed that when liver phospholipids FA were estimated, the FA that contributed to the difference at most were PA (*p* < 0.01), OA (*p* < 0.05), ETA (*p* < 0.01), AA (*p* < 0.01), EPA (*p* < 0.001), DTA (*p* < 0.001), DPA (*p* < 0.05) and DHA (*p* < 0.01). For further analyses, FA were divided to: 1) saturated (SFA) and MUFA, and 2) PUFA. When SFA and MUFA were considered (Fig. 8), all analyzed FA contributed to the difference in composition. Analyzed groups are significantly distanced except males fed on standard diet and males fed on fish based diet. This means that these two types of diet are not significantly different in males regarding the composition of FA. Furthermore, the composition of FA in females is different from males. Similar results were obtained when PUFA were taken into consideration, although difference between males and females is not so obvious regarding position in canonical space (Fig. 9).

## Discussion

Our results showed that each type of diet changes the composition of lipids and their ratio in the liver of rats. However, effects were not same for both sexes; particular type of diet led to different changes of lipid composition in different sexes. Furthermore, initial difference between sexes can be modulated by the type of diet.

Milk-based diet significantly elevated ALT and AST activities in males comparing to standard diet, while fish based diet had no effects in both females and males. In line with our results, no effect on the activity of these enzymes in healthy albino female rats treated with cow and camel milk was found [13]. On the other side, AST activity decreased, and ALT remained unchanged in camel milk treated male rats with hepatocarcinoma [14]. Regarding fish based diet Popovic et al. [6] showed that fish oil treatment did not change ALT and AST activities in male Wistar rats during 6 weeks.

In young Wistar rats treatment with fish oil decreased plasma TG and LDL cholesterol and increased HDL cholesterol concentration which is in line with our results [6]. Berge et al. [15] in their study in vitro showed that more EPA than DHA decresed concentration of TG in plasma. Rats treated with EPA and DHA esters for 3 months had at the end of study decreased concentration of plasma cholesterol. Inhibition of HMG-CoA activity in DHA-EE (ester) treated rats could have hypocholesterolemic effect [16].

Alabdulkarim B. [13] detected the decrease of total cholesterol and TG level in rats fed on 100% cow’s milk. Similarly, TG level was lower in standard diet-fed compared to fasted mice [17]. Decreasing tendency of serum TG, concomitant with increase of HDL and reduction of LDL/HDL was also found in healthy humans fed cow milk diet, but only when milk fat content modified towards lower SFA and higher USFA. There are studies identifying regular milk fat as a potentially cholesterol-elevating, mostly due to SFA and cholesterol in its content [18]. According to our knowledge there is not enough literature data for female Wistar rats regarding blood lipid profile after dietary treatment based on milk.

Membrane fluidity depends on FA phospholipids profiles, FA lenght and unsaturation, althought more SFA in cell membrane decrease membrane fluidity [19]. Diet induced FA profile influence membrane FA and membrane protein function [20]. Diet changes the ratio of SFA, MUFA and PUFA but the effects were sex specific. Our study showed that milk based diet lowered SFA and elevated MUFA in males, and PUFA, in females comparing to standard diet. Considering SFA females had lower levels of PA (16:0), but increased levels of SA (18:0) comparing to males. Females had significantly higher levels of estimated activity of elongase 6 comparing to males, which is in consistence with literature data. Namely, Marks et al. [21] stated that females had increased expression of hepatic elongase 6, which appears to be mediated by sex hormones. As a consequence concentration of PA was lower, and concentration of SA was higher in PL in livers from female rats compared with males.

Our results showed that in male rats, milk-based diet lowered the amount of SFA, mostly due to reduction in SA (18:0). Same result was reported in intervention trail on healthy male subject, where the diet enriched with high-SFA dairy lipids altered all SFA measured in red blood cell membrane, with only SA being significantly lower [22]. Desaturase 9 activity was generally higher in males than in females. This is in consistence with the results that before any dietary intervention female rats had significantly lower level of palmitoleic (16:1 n-7) and oleic (18:1 n-9) MUFA. Similarly, Marks et al. [21] showed that females had significantly lower concentrations of MUFA in liver and plasma PL which is largely attributable to differences in OA concentrations, but females also exhibited lower palmitoleic acid in plasma PL as well as lower VA (18:1 n-7) in liver phospholipids. In our study, in females milk based diet elevated OA content, while in males the same diet elevated overall MUFA. In the study of Poppitt et al. [22] diet enriched with high-USFA dairy lipids elevated MUFA OA (18:1n-9), what is in line with our results. In addition, the level of OA in blood and tissue is more dependent on endogenous metabolism than on dietary intake [23].

Diet based on both fish and milk lowered the amount of VA in liver PL of females, while in males, milk based diet elevated the amount of VA. The physiologic role of VA has yet to be elucidated. Recent studies have shown that VA could represent a potential marker of various diseases such as chronic kidney disease, hypertension or heart failure [24]. Ma et al. [25] demonstrated that VA was positively correlated with lower diabetes risk. According to our results, female rats had lower level of ETA (20:3 n-6) than males, at the beginning of the study. Lower ETA in liver PL of female rats is in correlation with results of Onozato et al. [26] who supposed that there may be sex-based differences in the biosynthetic production or metabolic processes of γ-linolenic acid and ETA in human. In females, fish and milk based diet elevated ETA level comparing to standard diet. Females fed with standard diet had the lowest level of desaturase 6, but feeding with milk and fish based diet elevated its activity almost to the levels measured in males. Literature data suggest that the higher level of ETA could be beneficial for human health. Namely, ETA can be converted to prostaglandins of the 1-series (PGE1) which have been found to exert clinical efficacy in a variety of diseases, including suppression of chronic inflammation, vasodilatation and lowering of blood pressure [27]. Generally speaking, the most pronounced effect of different types of food was noted in PUFA, with milk and fish based diet elicited mostly opposite effect. Considering our results, significant modulatory effect of milk-based diet was found. In males, milk based diet decreased EPA (20:5 n-3), DHA (22:6 n-3) and total n-3 and increased LA (18:2 n-6), DTA (22:4 n-6), n-6 and n-6/n-3 ratio comparing to the standard and fish-based diet. Unlike milk-based diet, fish-based diet elevated only DHA levels in males. In line with our results Poppitt et al. [22] reported decrease of total n-3, EPA and DHA in humans on dairy lipids-enriched diets. On the other side, in the study of Oarada et al. [28] after receiving EPA and DHA, these FA increased in plasma PL of male mice, while AA (20:4 n-6) and n-6/n-3 ratio decreased. Also, treatment with fish oil increased total n-3, EPA, DPA (22:5 n-3) and LA, and decreased total n-6, AA and n-6/n-3 ratio in liver PL of young and aged Wistar rats [6, 29, 30]. Our female rats treated with fishmeal showed increased n-3 and decreased n-6 and n-6/n-3 ratio. In terms of the individual FA, the decrease of n-6 LA and AA and elevation of n-3 EPA, DPA and DHA was observed. Increase of EPA despite reduced Δ5 desaturase activity in females fed on fish based diet, most likely comes from significant amount of EPA in fish-based diet. A significant sex dependent change was observed in plasma phosphatidyl choline LA, EPA, DPA, and total long chain n-3 PUFA concentrations after fish oil treatment in human with significant dose response to fish oil evident only in females [31]. Interestingly, Walker et al. [32] identified small sex differences in incorporation EPA and DHA, after supplementation, in different lipid classes of plasma, cells and tissues. In general, n-6 are known to promote inflammation, serving as precursors of inflammatory factors and up-regulators of various genes of inflammatory signalling. Precisely, arachidonic acid, present in different tissue, can be converted in pro-inflammatory eicosanoids connected with inflammatory processes and some chronic diseases. Also, higher n-6/n-3 ratio hinders structure and activity of cell membranes [33]. Linoleic acid could down-regulate n-3 PUFA desaturation, resulting in their loss from membrane phospholipids [34]. In contrast, n-3, particularly EPA and DHA, are competitive substrates for the same enzymes as AA, they antagonize the pro-inflammatory effects of n-6 and improve membrane morphology [33]. Besides being precursor of anti-inflammatory factors, EPA partially blocks conversion of AA and DTA to harmful eicosanoids and reduce cardiovascular risk and tumour growth [35, 36]. DHA has protective role in Alzheimer disease and some type of dementia [37]. Several studies showed inverted correlation between overall n-3 in serum or plasma PL and cardiovascular risk and atherosclerosis [38–40]. Considering all above, lower EPA and DHA and elevated n-6/n-3 ratio in liver phospholipids of males fed on milk based diet, made us to assume about possible higher inflammation susceptibility and cell vulnerability in our milk based diet-fed males. In opposite, elevated long-chain n-3 PUFA and decreased n-6/n-3 ratio in females fed on fish based diet speaks in favour of improved FA profile and possible health benefits in females due to fish-based diet. In addition, possible epigenetic effects of applied diets could not be excluded, since maternal fat intake has already been found to alter FA proportions in the offspring [41, 42].

Since type of diet significantly changes the percentage of individual FA in liver PL, the significance of these changes for FA composition for particular type of diet was tested by canonical discriminant analysis. Canonical discriminant analysis showed that FA that mostly contributed to the difference were PA (16:0), OA (18:1 n-9), ETA (20:3 n-6), AA (20:4 n-6), EPA (20:5 n-3), DTA (22:4 n-6), DPA (22:5 n-3) and DHA (22:6 n-3). For further analyses, FA were divided into: 1) SFA and MUFA, and 2) PUFA. Canonical discriminant analyses showed that SFA and MUFA composition in liver PL of males fed on standard and fish based diet were not significantly different. Similar results were obtained when total PUFA were taken into consideration, although difference between males and females is not so obvious regarding position in canonical space.

## Conclusions

In conclusion, gender related variations in FA composition of rat liver PL were observed, and our results have shown that those initial differences can be significantly modulated by the type of diet. Furthermore, the modulatory effects of milk- and fish-based diets on liver phospholipids FA profiles appeared to be sex-specific. When FA of liver PL were observed, the FA that mostly contributed to the difference were PA, OA, ETA, AA, EPA, DTA, DPA and DHA. Considering type of diet, fish based diet appears to exert more beneficial response relating individual FA composition, with more pronounced effects in females indicating that gender differences should be considered for nutritional recommendation in human diets.

## Abbreviations

AA: Arachidonic acid (C20:4 n-6)
ALT: Alanine aminotransferase
ANOVA: two-way analysis of variance
AST: Aspartate aminotransferase
DHA: Docosahexaenoic acid (C22:6 n-3)
DPA: Docosapentaenoic acid (C22:5 n-3)
DTA: Docosatetraenoic acid (C22:4 n-6)
EPA: Eicosapentaenoic acid (C20:5 n-3)
ETA: Eicosatrienoic acid (C20:3 n-6)
FA: Fatty acids
FF: females fed on fish based diet
FM: females fed on milk based diet
FS: females fed on standard diet
HDL: High-density lipoproteins
LA: Linoleic acids (C18:2 n-6)
LDL: Low-density lipoproteins
MF: males fed on fish based diet
MM: males fed on milk based diet
MS: males fed on standard diet
MUFA: Monounsaturated fatty acids
OA: Oleic acid (C18:1 n-9)
PA: Palmitic acid (C16:0)
PL: Phospholipids
PUFA: Polyunsaturated fatty acids
SA: Stearic acid (C18:0)
SFA: Saturated fatty acids
TG: Triglycerides
VA: Vaccenic acid (C18:1 n-7)

## References

[1] Nguyen P, Leray V, Diez M, Serisier S, Le Bloch J, Siliart B, Dumon H (2008). Liver lipid metabolism. J Anim Physiol Anim Nutr.

[2] Oguzhan B, Sancho V, Acitores A, Villanueva-Peñacarrillo ML, Portois L, Chardigny JM, Sener A, Carpentier YA, Malaisse WJ (2006). Alteration of adipocyte metabolism in omega3 fatty acid-depleted rats. Horm Metab Res.

[3] Childs CE, Romeu-Nadal M, Burdge GC, Calder PC (2008). Gender differences in the n-3 fatty acid content of tissues. Proc Nutr Soc.

[4] Burdge GC, Calder PC (2005). Conversion of alpha-linolenic acid to longer-chain polyunsaturated fatty acids in human adults. Reprod Nutr Dev.

[5] Harris WS, Mozaffarian D, Lefevre M, Toner CD, Colombo J, Cunnane SC, Holden JM, Klurfeld DM, Morris MC, Whelan J (2009). Towards establishing dietary reference intakes for eicosapentaenoic and docosahexaenoic acids. J Nutr.

[6] Popović T, Borozan S, Arsić A, Martačić JD, Vučić V, Trbović A, Mandić L, Glibetić M (2012). Fish oil supplementation improved liver phospholipids fatty acid composition and parameters of oxidative stress in male Wistar rats. J Anim Physiol Anim Nutr.

[7] Zhou AL, Hintze KJ, Jimenez-Flores R, Ward RE (2012). Dietary fat composition influences tissue lipid profile and gene expression in Fischer-344 rats. Lipids.

[8] Burdge GC, Slater-Jefferies JL, Grant RA, Chung WS, West AL, Lillycrop KA, Hanson MA, Calder PC (2008). Sex, but not maternal protein or folic acid intake, determines the fatty acid composition of hepatic phospholipids, but not of triacylglycerol, in adult rats. Prostaglandins Leukot Essent Fatty Acids.

[9] Extier A, Langelier B, Perruchot MH, Guesnet P, Van Veldhoven PP, Lavialle M, Alessandri JM (2010). Gender affects liver desaturase expression in a rat model of n-3 fatty acid repletion. J Nutr Biochem.

[10] Kitson AP, Smith TL, Marks KA, Stark KD (2012). Tissue-specific sex differences in docosahexaenoic acid and Δ6-desaturase in rats fed a standard chow diet. Appl Physiol Nutr Metab.

[11] Hinkle ED, Wiersma W, Jurs GS (2003). Applied statistics for behavioral sciences.

[12] Manly BFJ (1986). Multivariate statistical methods: a primer.

[13] Alabdulkarim B (2012). Effect of camel milk on blood glucose, cholesterol, triglyceride and liver enzymes activities in female albino rats. World App Sci J.

[14] El Miniawy HMF, Ahmed KA, Tony MA, Mansour SA, Salah Khattab MM (2014). Camel milk inhibits murine hepatic carcinogenesis, initiated by diethylnitrosamine and promotedbyphenobarbitone. Int J Vet Sci Med.

[15] Berge RK, Madsen L, Vaagenes H, Tronstad KJ, Gottlicher M, Rustan AC (1999). In contrast with docosahexaenoic acid, eicosapentaenoic acid and hypolipidaemic derivatives decrease hepatic synthesis and secretion of triacylglycerol by decreased diacylglycerolacyltransferase activity and stimulation of fatty acid oxidation. Biochem J.

[16] Froyland L, Vaagenes H, Asiedu DK, Garras A, Lie O, Berge RK (1996). Chronic administration of eicosapentaenoic acid and docosahexaenoic acid as ethyl esters reduced plasma cholesterol and changed the fatty acid composition in rat blood and organs. Lipids.

[17] Gu Q, Yang X, Lin L, Li S, Li Q, Zhong S, Peng J, Cui Z (2014). Genetic ablation of solute carrier family 7a3a leads to hepatic steatosis in zebrafish during fasting. Hepatology.

[18] Ney DM (1991). Potential for enhancing the nutritional properties of milk fat. J Dairy Sci.

[19] Popovic T, Ranic M, Bulajic P, Milicevic M, Arsic A, Vucic V, Glibetic M (2009). Effects of n-3 fatty acids supplementation on plasma phospholipids fatty acid composition in patients with obstructive jaundice- a pilot study. J Clin Biochem Nutr.

[20] Hynes GR, Heshka J, Chadee K, Jones PJ (2003). Effects of dietary fat type and energy restriction on adipose tissue fatty acid composition and leptin production in rats. J Lipid Res.

[21] Marks KA, Kitson AP, Stark KD (2013). Hepatic and plasma sex differences in saturated and monounsaturated fatty acids are associated with differences in expression of elongase 6, but not stearoyl-CoA desaturase in Sprague-Dawley rats. Genes Nutr.

[22] Poppitt SD, Kilmartin P, Butler P, Keogh GF (2005). Assessment of erythrocyte phospholipid fatty acid composition as a biomarker for dietary MUFA, PUFA or saturated fatty acid intake in a controlled cross-over intervention trial. Lipids Health Dis.

[23] de Lorgeril M, Salen P (2012). New insights into the health effects of dietarysaturated and omega-6 and omega-3 polyunsaturated fatty acids. BMC Med.

[24] Pédrono F, Boulier-Monthéan N, Catheline D, Legrand P (2015). Impact of a standard rodent chow diet on tissue n-6 fatty acids, Δ9-Desaturation index, and Plasmalogen mass in rats fed for one year. Lipids.

[25] Ma W, Wu JH, Wang Q, Lemaitre RN, Mukamal KJ, Djoussé L, King IB, Song X, Biggs ML, Delaney JA (2015). Prospective association of fatty acids in the de novo lipogenesis pathway with risk of type 2 diabetes: the cardiovascular health study. Am J Clin Nutr.

[26] Onozato M, Nishikiori M, Iizuka H, Ichiba H, Sadamoto K, Fukushima T (2015). Determination of sex-based differences in serum ã-linoleic acid and dihomo-ã-linoleic acid using gas chromatography-mass spectrometry. J Chromatogr B Analyt Technol Biomed Life Sci.

[27] Wang X, Lin H, Gu Y. Multiple roles of dihomo-γ-linolenic acid against proliferation diseases. Lipids Health Dis. 2012; doi:10.1186/1476-511X-11-25. 10.1186/1476-511X-11-25 PMC3295719 22333072

[28] Oarada M, Furukawa H, Majima T, Miyazawa T (2000). Fish oil diet affects on oxidative senescence of red blood cells linked to degeneration of spleen cells in mice. Biochim Biophys Acta.

[29] Popović T, Borozan S, Arsić A, Debeljak-Martačić J, Vučić V, de Luka S, Milovanović I, Trbović A, Glibetić M (2011). Effects of n–3 supplementation on plasma and liver Phospholipid fatty acids profile in aged Wistar rats. Croat Chem Acta.

[30] Popović TB, Borozan SZ, Takić MM, Kojadinović MJ, Rankovic S, Ranić M, de Luka SR (2014). Fatty acid composition and oxidative stress parameters in plasma after fish oil supplementation in aging. Croat ChemActa.

[31] Caslake MJ, Miles EA, Kofler BM, Lietz G, Curtis P, Armah CK, Kimber AC, Grew JP, Farrell L, Stannard J (2008). Effect of sex and genotype on cardiovascular biomarker response to fish oils: the FINGEN study. Am J Clin Nutr.

[32] Walker CG, Browning LM, Mander AP, Madden J, West AL, Calder PC, Jebb SA (2014). Age and sex differences in the incorporation of EPA and DHA into plasma fractions, cells and adipose tissue in humans. Br J Nutr.

[33] Schmitz G, Ecker J (2008). The opposing effects of n-3 and n-6 fatty acids. Prog Lipid Res.

[34] Petrović S, Arsić A, Debeljak-Martačić J, Đurendić-Brenesel M, Pilija V, Milić N, Popović T (2015). Effects of dietary supplementation with a mixture of buckwheat leaf and flower on fatty acid composition of rat brain phospholipids. Acta Vet.

[35] Bagga D, Wang L, Farias-Eisner R, Glaspy JA, Reddy ST (2003). Differential effects of prostaglandin derived from omega-6 and omega-3 polyunsaturated fatty acids on COX-2 expression and IL-6 secretion. Proc Nati Acad Sci U S A.

[36] Arsić A, Prekajski N, Vučić V, Tepšić J, Popović T, Vrvić M, Glibetić M (2009). Milk in human nutrition: comparison of fatty acid profiles. Acta Vet.

[37] Arterburn LM, Hall EB, Oken H (2006). Distribution, interconversion, and dose response of n-3 fatty acids in humans. Am J Clin Nutr.

[38] Simon JA, Hodgkins ML, Browner WS, Neuhaus JM, Bernert JT, Hulley SB (1995). Serum fatty acids and the risk of coronary heart disease. Am J Epidemiol.

[39] Lemaitre RN, King IB, Mozaffarian D, Kuller LH, Tracy RP, Siscovick DS (2003). N-3 polyunsaturated fatty acids, fatal ischemic heart disease, and nonfatal myocardial infarction in older adults: the cardiovascular health study. Am J Clin Nutr.

[40] Holub DJ, Holub BJ (2004). Omega-3 fatty acids from fish oils and cardiovascular disease. Mol Cell Biochem.

[41] Kelsall CJ, Hoile SP, Irvine NA, Masoodi M, Torrens C, Lillycrop KA, Calder PC, Clough GF, Hanson MA, Burdge GC (2012). Vascular dysfunction induced in offspring by maternal dietary fat involves altered arterial polyunsaturated fatty acid biosynthesis. PLoS One.

[42] Hoile SP, Irvine NA, Kelsall CJ, Sibbons C, Feunteun A, Collister A, Torrens C, Calder PC, Hanson MA, Lillycrp KA, Burdge GC (2013). Maternal fat intake in rats alters 20:4n-6 and 22:6n-3 status and the epigenetic regulation of Fads2 in offspring liver. J Nutr Biochem.

